# MafA Is Required for Postnatal Proliferation of Pancreatic β-Cells

**DOI:** 10.1371/journal.pone.0104184

**Published:** 2014-08-15

**Authors:** Koki Eto, Wataru Nishimura, Hisashi Oishi, Haruhide Udagawa, Miho Kawaguchi, Masaki Hiramoto, Toshiyoshi Fujiwara, Satoru Takahashi, Kazuki Yasuda

**Affiliations:** 1 Department of Metabolic Disorders, Diabetes Research Center, Research Institute, National Center for Global Health and Medicine, Tokyo, Japan; 2 Department of Gastroenterological Surgery, Okayama University Graduate School of Medicine, Dentistry and Pharmaceutical Sciences, Okayama, Japan; 3 Department of Anatomy and Embryology, Faculty of Medicine, University of Tsukuba, Tennodai, Tsukuba, Ibaraki, Japan; University of Lille Nord de France, France

## Abstract

The postnatal proliferation and maturation of insulin-secreting pancreatic β-cells are critical for glucose metabolism and disease development in adults. Elucidation of the molecular mechanisms underlying these events will be beneficial to direct the differentiation of stem cells into functional β-cells. Maturation of β-cells is accompanied by increased expression of MafA, an insulin gene transcription factor. Transcriptome analysis of *MafA* knockout islets revealed MafA is required for the expression of several molecules critical for β-cell function, including *Glut2*, *ZnT8*, *Granuphilin*, *Vdr*, *Pcsk1* and *Urocortin 3*, as well as *Prolactin receptor* (*Prlr*) and its downstream target *Cyclin D2* (*Ccnd2*). Inhibition of *MafA* expression in mouse islets or β-cell lines resulted in reduced expression of Prlr and Ccnd2, and MafA transactivated the *Prlr* promoter. Stimulation of β-cells by prolactin resulted in the phosphorylation and translocation of Stat5B and an increased nuclear pool of Ccnd2 via Prlr and Jak2. Consistent with these results, the loss of MafA resulted in impaired proliferation of β-cells at 4 weeks of age. These results suggest that MafA regulates the postnatal proliferation of β-cells via prolactin signaling.

## Introduction

Accumulating evidence suggests that postnatal organ development and maturation are critical for future health, especially with respect to metabolic disease [1]. Pancreatic β-cells vigorously proliferate postnatally to increase insulin secretion capacity [2], which is implicated in adult β-cell mass [3]. Although the compensatory growth of β-cell mass in insulin resistance has been intensively investigated [4], the signaling pathway that regulates postnatal proliferation of β-cells is less well known [5]. Uncovering this mechanism will elucidate how β-cell mass is regulated during development and how the insulin-expressing cells that differentiate from stem cells acquire the capacity to proliferate.

During gestation, prolactin signaling is involved in the proliferation of β-cells. Generally, placental lactogen or prolactin binds to prolactin receptor (Prlr), which phosphorylates Janus kinase 2 (Jak2) and signal transducer and activator of transcription 5B (Stat5B) [5]. Phosphorylated Stat5B translocates into the nucleus and activates the transcription of its target genes by binding to GAS motifs, the Stat5 binding sequences [6]. The downstream targets of Prlr/Jak2/Stat5B signaling in β-cells include insulin, glucose transporter 2 (Glut2), glucokinase (Gck), tryptophan hydroxylase 1 (Tph1), cyclin D2 (Ccnd2) and Prlr [6], [7]. In addition, prolactin signaling may also be involved in the proliferation of β-cells after birth, as *Prlr* knockout (KO) neonates have reduced β-cell mass [8].

Maturation of β-cells occurs concurrently with the expression of v-maf musculoaponeurotic fibrosarcoma oncogene family protein A (MafA) [9], a transcription factor that regulates the expression of insulin via the C1-A2 elements of the insulin promoter [10]. In the pancreas, MafA is expressed exclusively in mature β-cells. Forced expression of MafA with Pdx1 and Ngn3 converts pancreatic acinar cells into insulin-secreting cells [11]. MafA expression is reduced in the β-cell with compromised function [12]. In the islets of the *MafA* knockout (KO) mice, the ratio of the β-cell mass to the α-cell mass is normal at birth; however, this ratio is reduced during the neonatal period [13], suggesting that MafA may be involved in regulation of the postnatal β-cell mass. Thus, the role of MafA in postnatal proliferation of β-cells was investigated in this study.

## Materials and Methods

### Mice

This study was carried out in strict accordance with the Fundamental Guidelines for Proper Conduct of Animal Experiment and Related Activities in Academic Research Institutions under the jurisdiction of Ministry of Health, Labour and Walfare. The protocol was approved by the Animal Care and Use Committee of the National Center for Global Health and Medicine (Permission Number: 13104). Islet isolation and pancreatic dissection were performed under deep anesthesia followed by cervical dislocation, and all efforts were made to minimize suffering. The generation of *MafA* KO mice was described previously [13]. Male mice were analyzed in this study. Mice were genotyped by NaOH extraction methods as described previously [14]. The primers used in this analysis are listed in Table S2 in File S1.

### Construction of Mouse Prolactin Reporter Luciferase Vectors

A reporter vector containing the human *Prlr* promoter (*hPrlr*) was obtained from the Promoter Reporter GoClone Collection (Promega, Madison, WI). The mouse *Prlr* (*mPrlr*) luciferase reporter vectors *mPrlrP*-1, *mPrlrP*-2 and *mPrlrP*-3 were generated by amplifying 2359 bp, 1304 bp and 608 bp fragments of the *mPrlr* promoter from high-quality mouse genomic DNA (Clontech) by PCR with the primers listed in Table S3 in File S1. An in-fusion cloning kit (Promega) was utilized to clone the amplified products into the pGL4.10 vector (Clontech, Palo Alto, CA), which was digested with NheI and HindIII. The reporter vectors with deletions of the putative MafA binding regions, *mPrlrP*-5, *mPrlrP*-6, *mPrlrP*-8, *mPrlrP*-9 and *mPrlrP*-11, were generated using the PrimeSTAR Mutagenesis Basal Kit (Takara Bio, Shiga, Japan); the reactions were performed using the primers listed in Table S3 in File S1, and the pGL4.10-*mPrlrP*-1 reporter vector was used as a template. MafA binding sites were predicted with TRANSFAC (BIOBASE, Beverly, MA). The sequences of the reporter vectors were confirmed by sequencing with the universal RVprimer3.

### Transcriptome and Quantitative RT-PCR Analysis of Isolated Islets and Cultured Cells

The islets were isolated from *MafA* KO or wild-type mice at 7 weeks of age using collagenase digestion as described previously [14]. Total RNA was extracted from the isolated islets or cultured cells using the QIAshredder and RNeasy Micro Kit (Qiagen Valencia, CA) following the manufacturer's instructions. The concentration of purified RNA was measured by a NanoDrop ND 1000 Spectrophotometer (Thermo Scientific, Rockford, IL). The A260/280 of RNA from the wild-type and MafA KO islets were 1.94±0.07 and 1.86±0.04, respectively. RNA expression in the islets isolated from the *MafA* KO and wild-type mice was compared using the Mouse 430 2.0 Array (Affymetrix, Santa Clara, CA, USA, n = 2) as described previously [15]. Samples for the analysis were prepared in accordance with the manufacturer's protocol, and the results were analyzed using the DAVID 6.7 [16] and IPA programs (Ingenuity Systems, Redwood City, CA). Reverse transcription was performed using high-capacity cDNA reverse transcription kits (Applied Biosystems, Foster City, CA). Quantitative PCR amplification was performed using the TaqMan universal PCR master mix core reagent kit (Applied Biosystems) with the probes listed in Table S4 in File S1 and was analyzed using an ABI Prism 7900 (Applied Biosystems); C_t_ values were measured in duplicate. mRNA was quantified by normalization to β-actin expression using the 2-ΔΔCt method. For analysis of the islets from the *MafA* KO mice and wild-type littermates, the expression of *MafA* was examined in all assays to confirm that *MafA* was absent in KO islets. The data are presented as the means ± S.E.M., and statistical significance was determined using a two-tailed unpaired Student's *t*-test.

### Cell Culture

The rat β-cell line INS-1 [17], the mouse β-cell line MIN6 [18] and HeLa [19] cell lines were published previously. INS-1 cells were cultured in RPMI-1640 medium (Sigma, St. Louis, MO) supplemented with 10% (w/v) fetal bovine serum (FBS; Thermo Scientific), 1 mM sodium pyruvate, 10 mM HEPES, 100 U/ml penicillin, 100 mg/ml streptomycin and 55 µM β-mercaptoethanol (Life Technologies, Carlsbad, CA). MIN6 cells were cultured in Dulbecco's modified Eagle's medium (DMEM; Sigma) supplemented with 15% FBS, 100 U/ml penicillin, 100 mg/ml streptomycin and 55 µM β-mercaptoethanol. HeLa cells were cultured in DMEM supplemented with 10% FBS, 100 U/ml penicillin and 100 mg/ml streptomycin. All cell lines were cultured at 37°C with 5% CO_2_.

For the prolactin stimulation experiments, INS-1 cells were transfected with siRNA against mouse MafA, rat MafA or rat Prlr (Silencer Select siRNA s233236, s172995 or Silencer siRNA 48147, respectively) or control siRNA (Life Technologies) using Lipofectamine 2000 (Life Technologies) according to the manufacturer's protocol. The cells were plated in 6-well plates or 60 mm dishes, and the medium was changed 24 hours after transfection. The reduced expression of *MafA* or *Prlr* was confirmed by qRT-PCR 48 hours after transfection as described above. In parallel, the medium in the 60 mm dishes was changed to a defined serum-free medium consisting of RPMI-1640 medium supplemented with 10 mM glucose (Sigma), 0.1% human serum albumin, 10 µg/ml human transferrin, 0.1 nM triiodothyronine, 50 µM ethanolamine, 50 µM phosphoethanolamine (Wako, Osaka, Japan), 10 mM HEPES, 100 U/ml penicillin and 100 µg/ml streptomycin [20]. After 24 hours, mouse prolactin (R&D Systems, #1445-PL-050, Minneapolis, MN) was added at a final concentration of 1 µg/ml or at the indicated concentration, and the cells were incubated for 8 hours with or without AG490 (Millipore, Billerica, MA). The cells were then evaluated by immunoblot analysis or immunofluorescence.

### Reporter Assay

HeLa cells were transfected with the indicated reporter plasmids and the pCMV-β-gal plasmid (Promega), as an internal control, using Lipofectamine 2000 (Life Technologies) according to the manufacturer's protocol. The medium was changed 24 hours after transfection. At 48 hours after transfection, the firefly luciferase activity of the cells was analyzed using the luciferase assay system (Promega), and the β-gal activity was assessed as reported previously [14]. The data are presented as the means ± S.E.M., and statistical significance was determined using a two-tailed unpaired Student's *t*-test.

### Immunoblot Analysis

INS-1 cells were harvested in PBS and sonicated in 200 µl of buffer containing 10 mM Tris/HCl (pH 7.5), 150 mM NaCl, and 1% TX-100 supplemented with the protease inhibitor and phosphatase inhibitor cocktails (Nacalai Tesque, Kyoto, Japan). The lysates were centrifuged at 20,000×*g* rpm for 2 minutes. The supernatant was analyzed by SDS-PAGE followed by immunoblot analysis, as described previously [21]. For the nuclear fractionation, Subcellular Protein Fractionation Kit for Cultured Cells (Thermo Scientific) was used following the manufacturer's instructions. For immunoprecipitation, the supernatant was incubated with 20 µl of the indicated antibodies immobilized on 10 µl of protein G Sepharose 4 fast-flow beads (GE Healthcare, Fairfield, CT) for 3 hours at 4°C. After the beads were washed four times with 500 µl of buffer, containing 10 mM Tris/HCl (pH 7.5), 150 mM NaCl, and 0.33% Triton X-100 supplemented with protease inhibitor and phosphatase inhibitor cocktails, the immunoprecipitates were subjected to SDS-PAGE and immunoblot analysis with the antibodies listed in Table S5 in File S1. Images were obtained with ChemiDoc XRS Plus (Biorad, Hercules, CA) and quantified using Image Lab 3.0 (Biorad). The data are presented as the means ± S.E.M., and statistical significance was determined using a two-tailed unpaired Student's *t*-test.

### Immunofluorescence

For immunofluorescence of cells, INS-1 cells were fixed with 4% (w/v) paraformaldehyde for 15 min and were permeabilized with 0.3% (w/v) Triton X-100 in PBS for 15 min. After blocking with PBS containing 10% (w/v) BSA for 30 min, the cells were incubated overnight with the primary antibodies (Table S5 in File S1) in PBS containing 3% (w/v) BSA. The cells were then washed with PBS and incubated with Texas red-conjugated anti-rabbit IgG and FITC-conjugated anti-mouse IgG (Jackson ImmunoResearch, West Grove, PA) in PBS containing 3% (w/v) BSA for 1 h, followed by washing with PBS and mounting with Mounting Medium containing 4′,6-diamidino-2-phenylindole (DAPI) (Vector, Burlingame, CA).

Immunostaining analyses of mice pancreatic sections were performed on paraffin-embedded sections as described previously [14]. The primary antibodies used in this study are listed in Table S5 in File S1. For amplification, biotinylated anti-mouse antibodies (Jackson ImmunoResearch) were used at a 1∶400 dilution, followed by incubation with streptavidin-conjugated Alexa Fluor 488 (1∶400) (Life Technologies). The secondary antibody was DyLight 594-conjugated anti-guinea pig IgG (Jackson ImmunoResearch). DAPI mounting medium (Vector) was used to label the nuclei.

For both staining, immunofluorescent images were obtained using an Olympus FV-1000 (Olympus) in confocal mode, and the acquired images were identically processed using Adobe Photoshop CS5.1. Quantification was performed using NIH ImageJ software. For the BrdU^+^/insulin^+^ cells from the *MafA* KO and wild-type pancreata, a total of 1706 and 1262 insulin^+^ cells, respectively, were counted from 3 mice of each genotype. The data are presented as the means ± S.E.M., and statistical significance was determined using a two-tailed unpaired Student's *t*-test.

### BrdU Incorporation Study

A 10 mg/ml solution of 5′-bromo-2′ deoxyuridine (BrdU, Sigma) in PBS was filter-sterilized and kept on ice. A dose of 100 mg BrdU/kg body weight was injected intraperitoneally into the mice, followed by *ad libitum* feeding for 24 hours. The mice were sacrificed, and BrdU incorporation in the pancreas was analyzed by immunofluorescence; a small section of the duodenum was used as a positive control for BrdU incorporation.

## Results

### MafA Is Involved in the Expression of Prolactin Receptor in β-Cells

**Figure 1:**
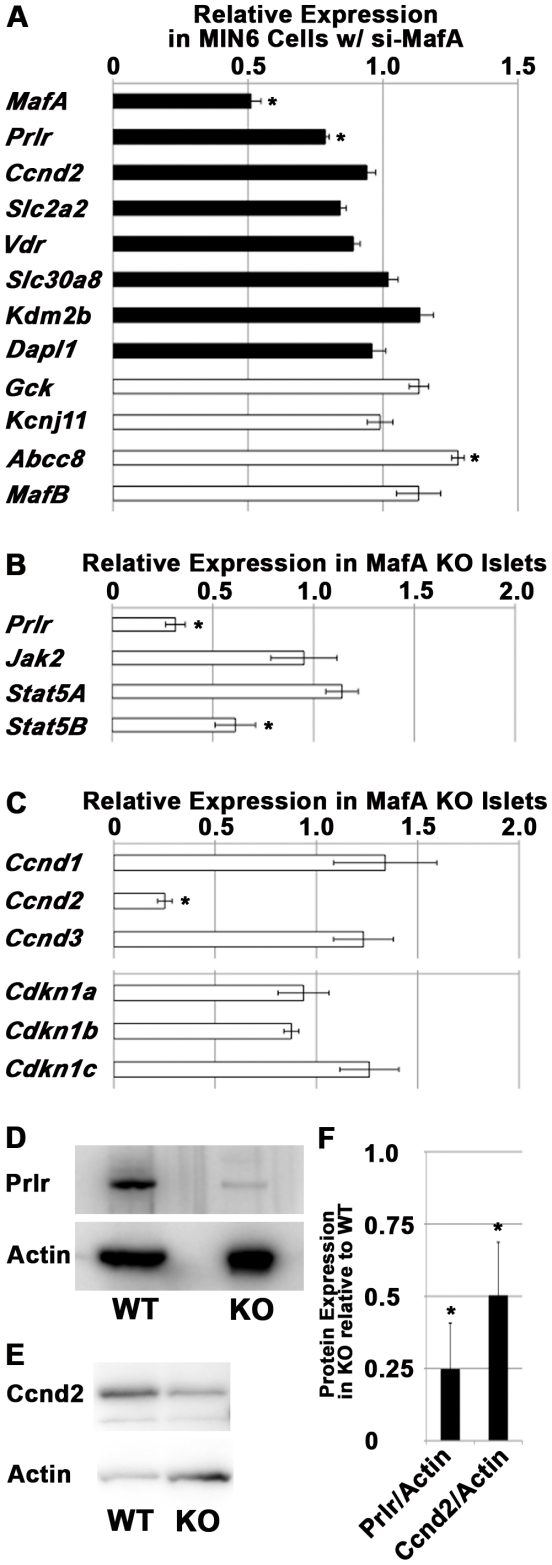
Inhibition of *MafA* expression in β-cell lines or mouse islets resulted in reduced expression of Prlr and Ccnd2. (A) mRNA expression of the indicated molecules in MIN6 cells transfected with *MafA* siRNA relative to cells transfected with control siRNA. n = 4 for each molecule, except n = 3 for *MafB*. Black bars: molecules detected in the transcriptome of *MafA* KO islets; white bars: molecules involved in β-cell function. (B) qRT-PCR analysis of prolactin signaling components in *MafA* KO islets relative to wild-type islets. n = 5 for Prlr; n = 3 for *Jak2*, *Stat5A* and *Stat5B*. (C) qRT-PCR analysis of molecules involved in the cell cycle in *MafA* KO islets relative to wild-type islets. n = 4 for *Ccnd1*; n = 5 for *Ccnd2* and *Ccnd3*; n = 3 for *Cdkn1a*, *Cdkn1b* and *Cdkn1c*. (D) Immunoblots of Prlr and actin in wild-type (WT) and *MafA* KO (KO) islets. n = 3. (E) Immunoblots of Ccnd2 and actin in wild-type (WT) and *MafA* KO (KO) islets. n = 3. (F) Quantification of the results of (D) and (E). The data represent the mean ± S.E.M. in (A), (B), (C) and (F), and representative results are shown in (D) and (E). Asterisks means p<0.05.

To examine the role of MafA in β-cell, transcriptome analysis of MafA KO islets at 7 weeks of age was performed. Several downregulated genes were detected, including *Slc30a8* (*ZnT8*), *Vdr*, *Slc2a2* (*Glut2*), *Pcsk1*, *Crystalline*, *Sytl4* (*Granuphilin*), *Urocortin 3*, *Prlr* and *Ccnd2* (Table S1 in File S1). Among the 9 molecules that were downregulated in both sets (Table 1), prolactin receptor (Prlr) was our primary focus because *Prlr* KO neonates have reduced β-cell mass [8]. The expression of *Ccnd2*, one of the downstream targets of prolactin signaling [22], was also markedly reduced in *MafA* KO islets (Table 1). Several genes that were downregulated in *MafA* KO islets, along with several molecules critical for β-cell function, were examined in mouse β-cell line MIN6 cells that were transfected with *MafA* siRNA and MIN6 cells that were transfected with control siRNA. While *MafA* was downregulated to 51.2±3.7% of its original expression (*p<0.01*), *Prlr* was significantly reduced to 78.6±1.5% relative to the controls (*p<0.05*; Fig. 1A). These results were further confirmed by analysis of the *MafA* KO islets by qRT-PCR. *Prlr* expression was reduced to 31.5±4.9% in the *MafA* KO islets compared to the wild-type islets at 7 weeks of age (*p<0.01*; Fig. 1B). The expression of the prolactin signaling components *Jak2* and *Stat5A* was not affected (*p>0.05*, respectively), while *Stat5B* expression was decreased (*p<0.01*). The expression of *Ccnd2* was markedly reduced to 25.2±3.6% (*p<0.01*), while *Ccnd1*, *Ccnd3*, *Cdkn1a*, *Cdkn1b* and *Cdkn1c* were not impaired in *MafA* KO islets compared with wild-type islets (*p>0.05*, respectively; Fig. 1C). Reduced protein expression of Prlr and Ccnd2 was also detected in *MafA* KO islets (Fig. 1D and E). Quantification and statistical analyses showed that the expression of Prlr and Ccnd2 in KO islets was 24.8±15.9% and 50.3±15.9% of those in wild-type islets (*p = 0.04 and 0.05*), respectively (Fig. 1F). These results suggest that MafA is directly or indirectly involved in the expression of Prlr and Ccnd2 in β-cells.

**Table 1:** Genes that were downregulated in MafA KO islets in both transcriptome datasets.

Probe set	Gene	Gene symbol	Fold increase (WT/KO)	
			Set 1	Set 2
1418783_at	Transient receptor potential cation channel, subfamily M, member 5	*Trpm5*	14.9	26.0
1443413_s_at			n.d.	21.1
1449224_at			n.d.	9.9
1434354_at	Monoamine oxidase B	*Maob*	8.6	7.0
1417336_a_at	Synaptotagmin-like 4/Granuphilin	*Sytl4*	8.0	14. 9
1422837_at	Sciellin	*Scel*	4.9	2.1
1441102_at	Prolactin receptor	*Prlr*	4.0	2.6
1448556_at			3.0	2.3
1437397_at			2.6	2.3
1425853_s_at			2.1	2.6
1454632_at	RIKEN cDNA 6330442E10 gene	*Tmem229B*	3.7	4.0
1457254_x_at			4.0	6.1
1424291_at	Similar to nucleoporin 93; nucleoporin 93	*Nup93*	4.0	2.1
1416123_at	Cyclin D2	*Ccnd2*	3.5	4.3
1448229_s_at			2.8	3.7
1430127_a_at			2.6	2.8
1455956_x_at			2.5	3.3
1416124_at			n.d.	2.6
1416122_at			2.1	2.5
1434745_at			2.0	2.3
1423640_at	Synaptoporin	*Synpr*	3.5	4.3

**Figure 2:**
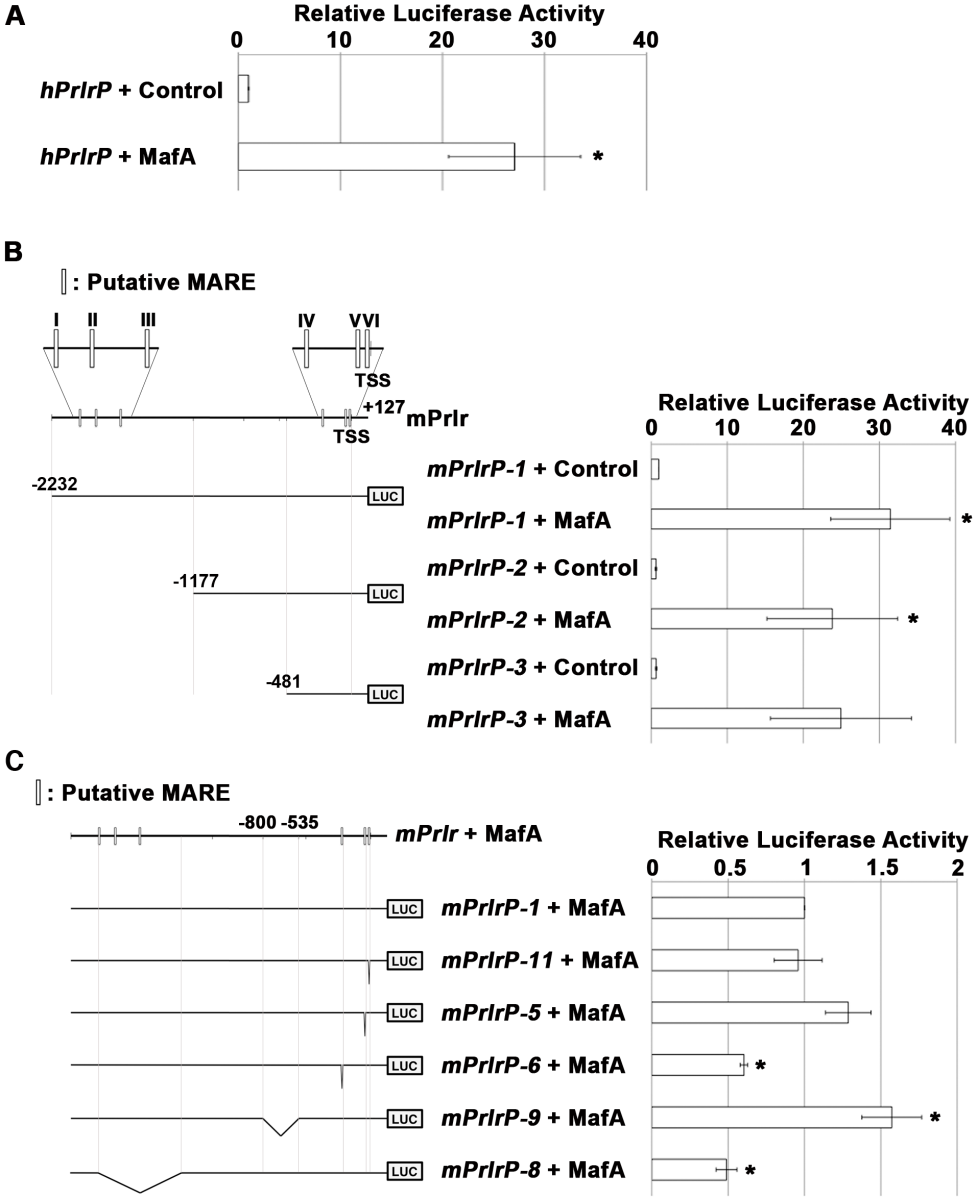
Activation of the *Prlr* promoter by MafA. The effects of MafA expression on *Prlr* promoter activity were analyzed using HeLa cells. (A) Luciferase activity driven by the *hPrlr* promoter (from −890 to +103) in MafA-overexpressing cells compared to control cells transfected with the control vector pcDNA3.1. n = 8. (B) Luciferase activities driven by the *mPrlr* promoter constructs, including −2232 to +127 (*mPrlrP*-1), −1177 to +127 (*mPrlrP*-2) and −481 to +127 (*mPrlrP*-3), with or without overexpression of MafA, relative to the luciferase activity of *mPrlrP*-1 with control. n = 5. Six putative MAREs were observed in the *mPrlr* promoter and are referred to as region I (from −2026 to −2016), region II (from −1907 to −1897), region III (from −1723 to −1713), region IV (from −217 to −207), region V (from −47 to −37) and region VI (from −12 to −2). TSS: transcription start site. (C) Luciferase activities of the *mPrlrP-1* construct (−2232 to +127) with MafA compared to the luciferase activities of constructs with deletions in the following regions: region VI (from −12 to −2, *mPlrlP*-11), region V (from −47 to −37, *mPlrlP*-5), region IV (from −217 to −207, *mPlrlP*-6), from −800 to −535 (*mPlrlP*-9) or regions I to III (from −2026 to −1409, *mPlrlP*-8). n = 4. The data represent the mean ± S.E.M. Asterisks means p<0.05.

*In silico* analysis detected six conserved MafA recognition elements (MAREs) between the transcription start site (TSS) and −3000 bp in the mouse *Prlr* promoter (Fig. 2B), and these sites were also preserved in the human and rat genes (data not shown). A luciferase reporter assay using the human and mouse *Prlr* (*hPrlr* and *mPrlr*, respectively) promoters clearly showed that overexpression of MafA transactivated both the *hPrlr* (*p<0.01*) and *mPrlr* (*p<0.05*) promoters in HeLa cells (Fig. 2A and B). MafA activated the −2232 to +127 *mPrlr* promoter fragment to a greater extent than the −1177 to +127 fragment (*p = 0.05*) and the −481 to +127 fragment (*p = 0.06*) (Fig. 2B). Moreover, the relative luciferase activity of various *mPrlr* promoter deletion constructs concomitant with forced expression of MafA revealed that the regions from −217 to −207 (IV; *p<0.01*) and from −2026 to −1409 (I to III; *p<0.01*) were involved in the MafA-mediated transcriptional activation of the *mPrlr* promoter (Fig. 2C). These results suggest that MafA transcriptionally regulates the expression of *Prlr*.

### Prolactin Signaling Regulates the Expression of Cyclin D2 in β-Cells

**Figure 3:**
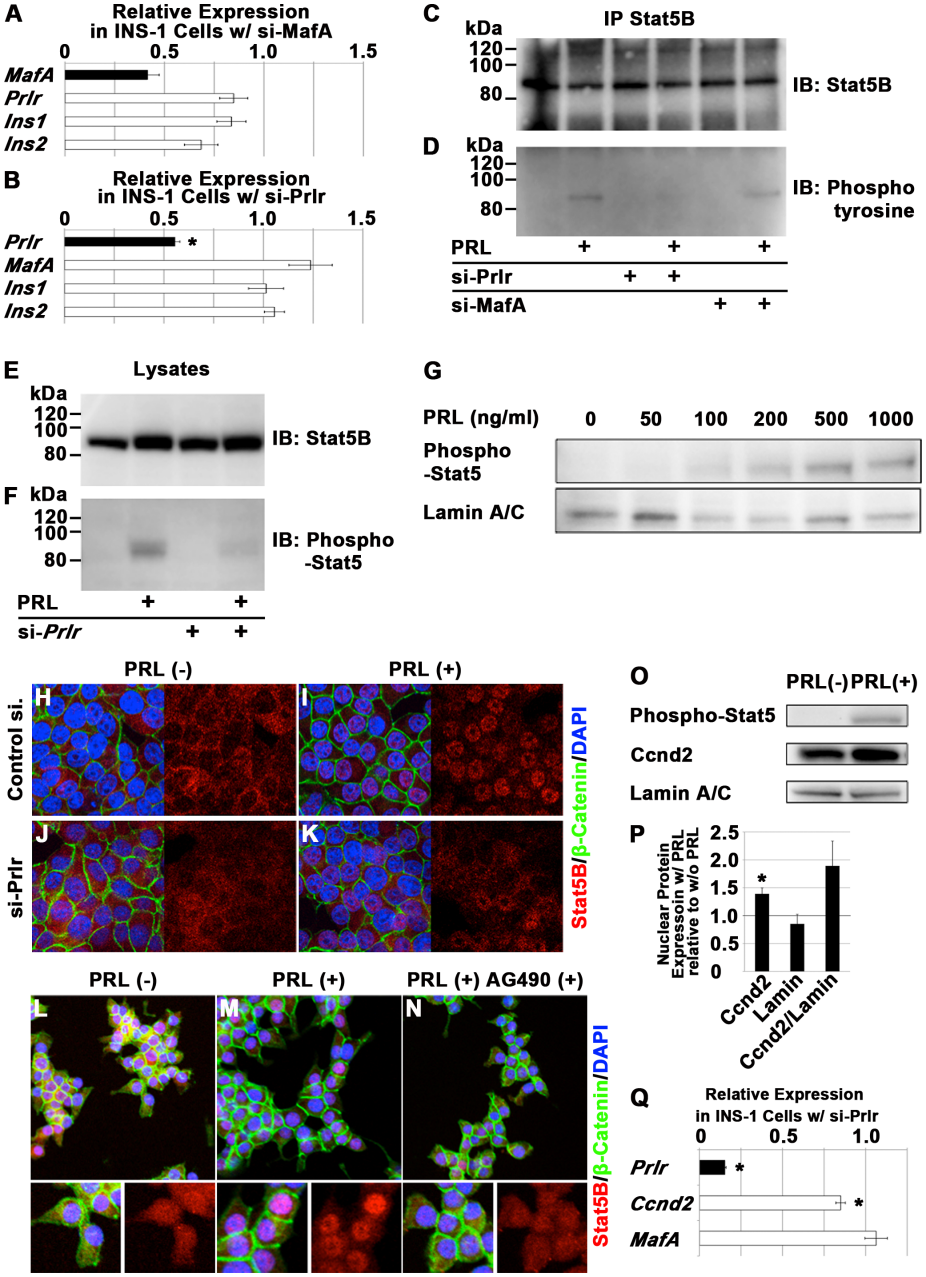
Regulation of Ccnd2 expression by prolactin signaling in β-cells. (A, B) mRNA expression of the indicated molecules in INS-1 cells transfected with siRNA targeting *MafA* (A) or *Prlr* (B) relative to INS-1 cells transfected with control siRNA. n = 4 for both (A) and (B). (C–P) INS-1 cells were transfected with control siRNA or siRNA targeting *Prlr* (si-*Prlr*) or *MafA* (si-*MafA*). At 48 hours after transfection, the cells were cultured in defined serum-free medium for 24 hours for serum deprivation, followed by the addition of prolactin (PRL) or PBS as a control. (C, D) The immunoprecipitates obtained with anti-Stat5B antibody from 1% TX-100 soluble fractions of INS-1 cells transfected with control si-RNA, si-*Prlr* or si-*MafA*, and with or without PRL stimulation were immunoblotted with either anti-Stat5B (C) or anti-phosphotyrosine antibody (D). n = 3. (E, F) 1% TX-100 soluble fractions of INS-1 cell lysates were immunoblotted with either anti-Stat5B (E) or anti-phosphorylated Stat5 antibody (F). n = 3. (G) Dose-dependent effects of PRL on the phosphorylation of Stat5. n = 2. (H–K) Localization of Stat5B (red), β-catenin (green) and nuclei, which were counterstained with DAPI (blue), in INS-1 cells that were transfected or not with si-*Prlr* and stimulated or not with PRL. n = 3. (L–N) Localization of Stat5B (red), β-catenin (green) and nuclei, which were counterstained with DAPI (blue), in INS-1 cells with or without PRL stimulation and with or without exposure to AG490, a Jak2 inhibitor. n = 3. (O, P) The amount of phosphorylated Stat5, Ccnd2 and Lamin A/C (control) in the nuclear fraction of INS-1 cells with (w/) or without (w/o) PRL stimulation after serum deprivation (O), and quantification of the results (P). n = 4. (Q) mRNA expression of the indicated molecules in INS-1 cells transfected with siRNA targeting *Prlr* relative to INS-1 cells transfected with control siRNA. n = 3 for each molecule. The data represent the mean ± S.E.M. in (A), (B), (P) and (Q), and representative results are shown in (C–O). Asterisks means p<0.05.

To determine whether MafA and Prlr are involved in the expression of Ccnd2 in β-cells, prolactin signaling was analyzed in rat β-cell line INS-1 cells, which were previously used to examine prolactin signaling [6], [7], [20], [23], [24]. The expression of MafA was 41.5±5.8% of the control level with transfection of siRNA targeting *MafA* into INS-1 cells (*p = 0.09*; Fig. 3A). Concomitant with the downregulation of *MafA*, the expression of *Prlr*, *Ins1* and *Ins2* was 84.8±7.0%, 83.7±7.3% and 68.5±8.4%, respectively, of the controls, but not statistically significant (*p>0.05*, respectively; Fig. 3A). Transfection of *Prlr* siRNA resulted in the reduction of *Prlr* expression to 55.7±2.3% of the control value (*p = 0.05*) without reducing the expression of *MafA*, *Ins1* or *Ins2* (Fig. 3B). In INS-1 cells, stimulation with 1 µg/ml prolactin resulted in the tyrosine phosphorylation of Stat5B (Fig. 3C–F), and this effect on Stat5B phosphorylation was dose-dependent (Fig. 3G). Inhibition of *Prlr* expression with siRNA resulted in reduced phosphorylation of Stat5B but did not affect its total protein level (Fig. 3C–F). However, Inhibition of *MafA* expression with siRNA, resulting in 15.2% reduction in *Prlr* expression (Fig. 3A), did not significantly alter the tyrosine phosphorylation of Stat5B in β-cells (Fig. 3C and D). Therefore, an siRNA targeting *Prlr* was used to examine the effect of reduced *Prlr* expression.

In INS-1 cells, the majority of Stat5B was localized in the cytoplasm (Fig. 3H) and was translocated into the nucleus after prolactin stimulation (Fig. 3I). However, this prolactin-induced redistribution of Stat5B was impaired when *Prlr* expression was inhibited by siRNA (Fig. 3J and K). Inhibition of the prolactin-induced translocation of Stat5B was also observed after the addition of AG490, a Jak2 inhibitor (Fig. 3L–N). These results confirm the previous findings that prolactin-induced phosphorylation and nuclear translocation of Stat5B are mediated by Prlr and Jak2 [20]. Using these systems, the effect of prolactin signaling on the expression of Ccnd2 was examined. With upregulation in the phosphorylation of Stat5 in INS-1 cells, prolactin stimulation increased the amount of nuclear Ccnd2 (Fig. 3O). Quantitative analysis demonstrated that nuclear Ccnd2 protein was increased to 139.2±10.6% after prolactin stimulation (*p = 0.01*). Ccnd2 protein relative to control Lamin A/C was 189.3±44.4% of the control level (*p = 0.09*; Fig. 3P). We also examined the mRNA expression of *Ccnd2* in INS-1 cells with siRNA targeting *Prlr* compared to those with control siRNA. mRNA expression of *Prlr* and *Ccnd2* in INS-1 cells with siRNA targeting *Prlr* was 15.3±0.5% (*p<0.01*) and 84.9±2.9% (*p = 0.01*) respectively, relative to the controls (Fig. 3Q). These results verify the previous data [7], [24], [25], suggesting that prolactin signaling regulates the expression of Ccnd2 in β-cells.

### Reduced Proliferation of β-Cells in *MafA* KO Mice at 4 Weeks of Age

**Figure 4:**
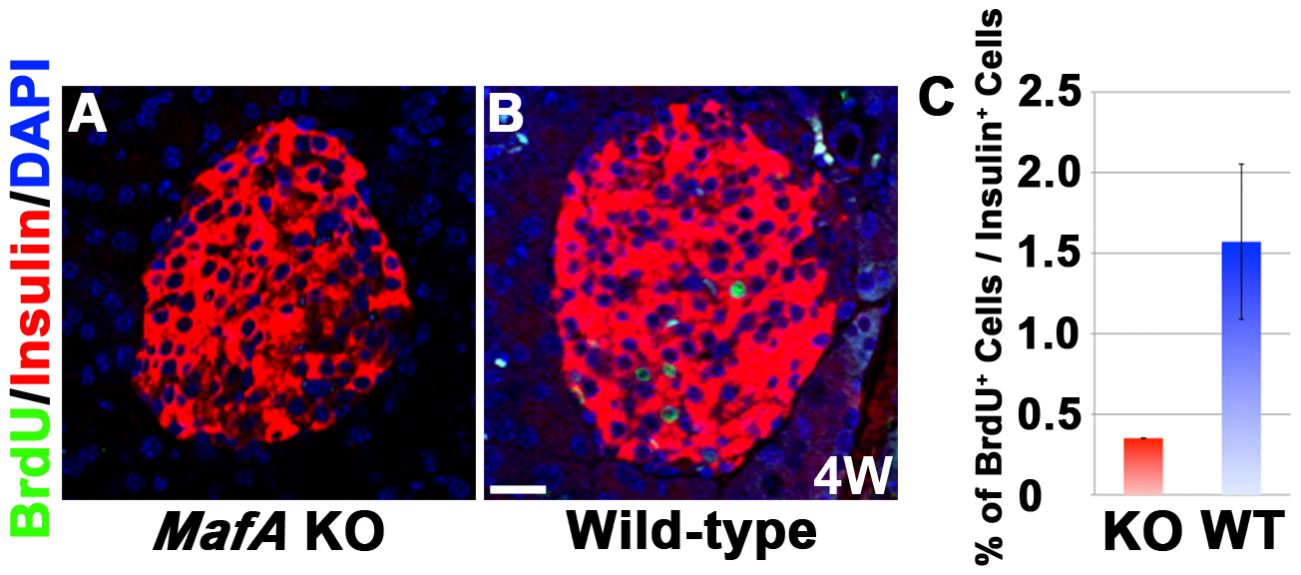
Impaired proliferation of β-cells in *MafA* KO mice. (A, B) Representative islets of *MafA* KO mice and their wild-type littermates 24 hours after the injection of BrdU (100 mg BrdU/kg body weight). BrdU (green), insulin (red) and DAPI (blue). (C) Quantification of the BrdU^+^ cells among the insulin^+^ cells. In total, 1706 insulin^+^ cells were counted in the *MafA* KO pancreas, and 1262 insulin^+^ cells were counted in the wild-type pancreas. The proliferation of β-cells in *MafA* KO mice was impaired. n = 3. The data represent the mean ± S.E.M. Bar, 20 µm.

Postnatal proliferation of β-cells is critical for the β-cell mass in adults [3]. Our above results prompted us to examine if the reduced expression of Prlr and Ccnd2 in the β-cells of *MafA* KO mice affected postnatal proliferation, as *Ccnd2* KO neonates exhibit reduced proliferation of β-cells and reduced β-cell mass [26]. The β-cell to α-cell ratio in the islets of the *MafA* KO mice is also reduced after birth [13]. A BrdU incorporation assay was performed in *MafA* KO mice and their wild-type littermates at 4 weeks of age. BrdU-positive staining was observed in 0.35±0.0% of β-cells in *MafA* KO pancreas, while 1.57±0.48% of β-cells in wild-type mice were BrdU-positive (p = 0.06; Fig. 4A–C). These results suggested that the loss of MafA impaired the proliferation of β-cells in 4-week-old mice. Collectively, these results reveal a new role for MafA in the postnatal proliferation of β-cells through its regulation of prolactin signaling.

## Discussion

This study described transcriptome analysis of the islets isolated from *MafA* KO mice. The results revealed the downstream candidates of MafA, and Prlr was a focus of this study. In the embryonic pancreas, Prlr is expressed primarily in acinar cells and ductal epithelium during early gestation. Later in gestation and in the postnatal period, Prlr is expressed predominantly in pancreatic islets [27], when MafA is expressed in β-cells [21]. The results from this study collectively suggest that MafA is critical for the expression of Prlr and that Prlr/Jak2/Stat5B signaling can induce the expression of Ccnd2 in β-cells. Consistent with these results, loss of MafA expression resulted in the impaired proliferation of postnatal β-cells. Thus, prolactin signaling may play an important role in the proliferation of neonatal β-cells under the control of MafA, in addition to its role in β-cell proliferation during gestation. Because the use of transformed β-cell lines may hamper the analysis of the promoter activity or the expression of Ccnd2 in detail, more analysis is needed to clarify the role of prolactin signaling on cell cycle in β-cells and to exclude the possibility that MafA directly activates the Ccnd2 promoter [28]. In addition to Prlr and the previously reported potential target genes of MafA/MafB, such as *ZnT8* [29], *Granuphilin* [30] and *Glut2* [13], [31], transcriptome analysis of the *MafA* KO islets in this study showed the downregulation of *Vdr*, *Pcsk1* and *Urocortin 3*, which are supposedly critical for β-cell function; however, the direct MafA binding sites in the promoters of these genes remain unknown.

In rodents, prolactin and placental lactogen bind only to Prlr [8]. During gestation, the expression of Prlr in pancreas and the serum prolactin level increase [5], [32], although the action of prolactin is antagonized by progesterone [33]. β-Cell-specific expression of placental lactogen-I results in accelerated β-cell proliferation, increased β-cell mass and number and increased insulin production, leading to hypoglycemia and elevated plasma insulin [34]. In contrast, *Prlr* KO neonates have reduced β-cell mass [8]. During pregnancy, *Prlr* ^+/−^ mice have reduced β-cell replication, but there is no increase in β-cell apoptosis, resulting in reduced β-cell mass. In *Prlr* ^+/−^ mice, impaired glucose clearance, decreased glucose-stimulated insulin release, higher post-prandial blood glucose, lower insulin levels and attenuated increases in islet density, β-cell number and mass are also observed throughout pregnancy, but not in the absence of pregnancy [35]. These results suggest the important role of Prlr in β-cell proliferation during pregnancy.

At birth, maternal serum prolactin continues to rise to facilitate mammary gland function, while secretion of placental lactogen from the placenta peaks during mid-gestation [5], [34]. Thus, prolactin, which may originate from the mother's feeding, may have a dominant effect on the neonatal proliferation of β-cells, not placental lactogen. An earlier study showed that prolactin and placental lactogen increase neonatal islet proliferation and insulin secretion [36]. Our results provide molecular evidence that Prlr is important for the postnatal proliferation of β-cells. Because β-cell replication in neonates plays a major role in β-cell mass in adult humans [3], the regulation of β-cell mass by prolactin signaling in postnatal pancreas may be implicated in an individual's susceptibility to diabetes.

The subcellular localization of Stat5 has been used to characterize the activation of the Jak2/Stat5 pathway by prolactin [20]. In β-cells, after exposure to prolactin, the redistribution of Stat5B from the cytoplasm to the nucleus was much higher than the redistribution of Stat5A, indicating that Stat5B plays a major role [23]. Stat5B binds to the GAS motif to induce the expression of its target genes [6], and our data suggested that Ccnd2 can be a Stat5B target gene. Consistent with our data, dominant-negative Stat5 reduces mRNA and protein level of Ccnd2 and inhibits S-phase entry [24], [25]. Constitutively active form of Stat5B binds to the GAS motif in the *Ccnd2* promoter, transactivates the *Ccnd2* promoter and induces the proliferation of β-cells [24]. Prolactin increases the mRNA expression of *Ccnd2* in rat islets [7]. *In vivo*, Ccnd2 is essential for the postnatal expansion of β-cell mass and the compensatory increase in β-cell mass in response to insulin-resistant states [20], [37]. However, during gestation or in neonates, the regulation of Ccnd2 expression by the Prlr/Jak2/Stat5B pathway and its role in the proliferation of β-cells have not been confirmed *in vivo* [5]. Indeed, in pregnant *Prlr* ^+/−^ mice, β-cell mass and Jak2 phosphorylation are decreased, but the expression of Ccnd2 is not changed [38], suggesting that more studies are necessary to investigate the precise role of prolactin signaling in the regulation of Ccnd2 expression and β-cell proliferation *in vivo*. Another target of prolactin signaling that may be implicated in β-cell proliferation is Tph1, an enzyme to synthesize serotonin [39]. However, recent study showed no difference in β-cell proliferation between Tph1 KO mice and wild-types during pregnancy even in the absence of serotonin [40], suggesting the importance of Ccnd2.

In addition to its effect on proliferation, accumulating evidence suggests that prolactin signaling is also critical for β-cell function. Prolactin increases the expression of molecules such as insulin, Glut2, Gck, Tph1, FoxM1 and Prlr in rat islets or INS-1 cells [6], [7]. Prolactin and placental lactogen stimulate insulin release and increase insulin content in cultured adult mouse islets and adult or newborn rat islets [41]. INS-1 cells constitutively expressing placental lactogen-II have increased *Preproinsulin* and *Glut2* mRNA [42]. These results raise the possibility that prolactin signaling is involved in the functional maturation of β-cells from the immature insulin-expressing cells found in neonates. Prolactin increases the binding of Stat5 to the GAS motif of the *Gck* promoter and the Gck synthesis in β-cells even in the absence of glucose [6], suggesting its action can be independent of glucose. It would be interesting to examine if the β-cell-specific expression of Prlr or Ccnd2 improves both the proliferation and function of β-cells in *MafA* KO mice. Although Bcl2 and BclXL are also downstream targets of prolactin signaling, there is no increase in the apoptosis rate of β-cells in pregnant *Prlr* ^+/−^ mice [35] and in *MafA* KO mice [43], suggesting that prolactin signaling does not play a major role in apoptosis of β-cells.

Clinical studies have demonstrated that men and women with hyperprolactinemia have postprandial hyperinsulinemia and an exaggerated insulin secretory response to glucose and arginine [44], [45]. Thus, further studies are needed to elucidate the effects of prolactin and placental lactogen on the proliferation and functional maturation of human β-cells. Moreover, activation of prolactin signaling or inhibition of progesterone signaling [33], [46] in insulin-expressing cells differentiated from human stem cells or endocrine precursor cells may enhance the proliferation and functional maturation of these cells.

## Supporting Information

File S1 **Tables S1–S5.** Table S1. Genes that were downregulated in the islets of *MafA* KO mice. Table S2. Genotyping primers used in this study. Table S3. Primers used to clone the indicated promoters or to mutagenize the *mPrlr* promoter. Table S4. TaqMan probes used in this study. Table S5. Antibodies used in this study.
(DOCX)
