# Correction: MafA Is Required for Postnatal Proliferation of Pancreatic β-Cells

**DOI:** 10.1371/journal.pone.0113892

**Published:** 2014-11-12

**Authors:** The PLOS ONE Staff

There is an error in Table 1. There are missing values in the Gene and Gene symbol columns due to incorrect formatting. Please see the corrected Table 1 here.

**Table 1:** Genes that were downregulated in MafA KO islets in both transcriptome datasets.

Probe set	Gene	Gene symbol	Fold increase (WT/KO)	
			Set 1	Set 2
1418783_at	Transient receptor potential cation channel, subfamily M, member 5	*Trpm5*	14.9	26.0
1443413_s_at	Transient receptor potential cation channel, subfamily M, member 5	*Trpm5*	n.d.	21.1
1449224_at	Transient receptor potential cation channel, subfamily M, member 5	*Trpm5*	n.d.	9.9
1434354_at	Monoamine oxidase B	*Maob*	8.6	7.0
1417336_a_at	Synaptotagmin-like 4 / Granuphilin	*Sytl4*	8.0	14. 9
1422837_at	Sciellin	*Scel*	4.9	2.1
1441102_at	Prolactin receptor	*Prlr*	4.0	2.6
1448556_at	Prolactin receptor	*Prlr*	3.0	2.3
1437397_at	Prolactin receptor	*Prlr*	2.6	2.3
1425853_s_at	Prolactin receptor	*Prlr*	2.1	2.6
1454632_at	RIKEN cDNA 6330442E10 gene	*Tmem229B*	3.7	4.0
1457254_x_at	RIKEN cDNA 6330442E10 gene	*Tmem229B*	4.0	6.1
1424291_at	Similar to nucleoporin 93; nucleoporin 93	*Nup93*	4.0	2.1
1416123_at	Cyclin D2	*Ccnd2*	3.5	4.3
1448229_s_at	Cyclin D2	*Ccnd2*	2.8	3.7
1430127_a_at	Cyclin D2	*Ccnd2*	2.6	2.8
1455956_x_at	Cyclin D2	*Ccnd2*	2.5	3.3
1416124_at	Cyclin D2	*Ccnd2*	n.d.	2.6
1416122_at	Cyclin D2	*Ccnd2*	2.1	2.5
1434745_at	Cyclin D2	*Ccnd2*	2.0	2.3
1423640_at	Synaptoporin	*Synpr*	3.5	4.3
