# Supplementary material for: MafA Is Required for Postnatal Proliferation of Pancreatic β-Cells
Source: PLoS One. 2014 Aug 15;9(8):e104184. doi: 10.1371/journal.pone.0104184 (PMC4134197; doi:10.1371/journal.pone.0104184)
Supplement: File S1 — Tables S1–S5. Table S1. Genes that were downregulated in the islets of MafA KO mice. Table S2. Genotyping primers used in this study. Table S3. Primers used to clone the indicated promoters or to mutagenize the mPrlr promoter. Table S4. TaqMan probes used in this study. Table S5. Antibodies used in this study. (DOCX) [file pone.0104184.s001.docx]

**MafA is required for postnatal proliferation of pancreatic β-cells**

Koki Eto, Wataru Nishimura, Hisashi Oishi, Haruhide Udagawa, Miho Kawaguchi, Masaki Hiramoto, Toshiyoshi Fujiwara, Satoru Takahashi and Kazuki Yasuda

**Supporting Information Legends**

Table S1-S5

**Table S1. Genes that were downregulated in the islets of *MafA* KO mice**

| **Probe set** | **Gene** | **Gene symbol** | **Fold increase (WT/KO)** | |
| --- | --- | --- | --- | --- |
|  |  |  | **Set 1** | **Set 2** |
| 1418783_at | transient receptor potential cation channel, subfamily M, member 5 | *Trpm5* | 14.9 | 26.0 |
| 1434354_at | monoamine oxidase B | *Maob* | 8.6 | 7.0 |
| 1424118_a_at | SPC25, NDC80 kinetochore complex component, homolog (S. cerevisiae) | *spc25* | 8.6 | n.d. |
| 1417336_a_at | synaptotagmin-like 4 / granuphilin | *Sytl4* | 8.0 | 14.9 |
| 1439816_at | predicted gene 10567 | *Gm10567* | 7.5 | n.d. |
| 1441991_at | cDNA sequence BC039632 | *BC039632* | 7.0 | n.d. |
| 1416561_at | glutamic acid decarboxylase 1 | *Gad1* | 6.5 | n.d. |
| 1435456_at | tetratricopeptide repeat domain 28 | *TTC28* | 6.1 | n.d. |
| 1417443_at | family with sequence similarity 151, member A | *Fam151a* | 5.7 | n.d. |
| 1419584_at | tetratricopeptide repeat domain 28 | *TTC28* | 5.7 | n.d. |
| 1453568_at | death-associated protein-like 1 | *Dapl1* | 5.3 | n.d. |
| 1422837_at | sciellin | *Scel* | 4.9 | 2.1 |
| 1436075_at | secreted frizzled-related sequence protein 5 | *Sfrp5* | 4.9 | n.d. |
| 1451478_at | angiopoietin-like 7 | *angptl7* | 4.6 | n.d. |
| 1418774_a_at | ATPase, Cu++ transporting, alpha polypeptide | *Atp7a* | 4.6 | n.d. |
| 1454984_at* | leukemia inhibitory factor receptor | *Lifr* | 4.6 | n.d. |
| 1448627_s_at | PDZ binding kinase | *Pbk* | 4.6 | n.d. |
| 1433653_at | family with sequence similarity 20, member A | *Fam20a* | 4.3 | n.d. |
| 1424547_at | carbonic anhydrase 10 | *Car10* | 4 | n.d. |
| 1451096_at | NADH dehydrogenase (ubiquinone) Fe-S protein 2 | *ndufs2* | 4 | n.d. |
| 1448552_s_at | prolactin receptor | *Prlr* | 4 | 2.6 |
| 1457254_x_at | RIKEN cDNA 6330442E10 gene | *Tmem229b* | 4 | 6.1 |
| 1424291_at | similar to nucleoporin 93; nucleoporin 93 | *Nup93* | 4 | 2.1 |
| 1448552_s_at | transmembrane protein 206 | *Tmem206* | 4 | n.d. |
| 1440888_at* | oxytocin receptor | *Oxtr* | 3.7 | n.d. |
| 1429206_at | Rho-related BTB domain containing 1 | *Rhobtb1* | 3.7 | n.d. |
| 1424131_at | collagen, type VI, alpha 3 | *Col6a3* | 3.5 | n.d. |
| 1416123_at | cyclin D2 | *Ccnd2* | 3.5 | 4.3 |
| 1417160_s_at | extracellular proteinase inhibitor | *expI* | 3.5 | n.d. |
| 1444027_at | solute carrier family 30 (zinc transporter), member 8 | *Slc30a8* | 3.5 | n.d. |
| 1423640_at | synaptoporin | *Synpr* | 3.5 | 4.3 |
| 1418176_at* | vitamin D receptor | *Vdr* | 3.5 | n.d. |
| 1423948_at | BCL2-associated athanogene 2 | *Bag2* | 3.3 | n.d. |
| 1452198_at* | lysine (K)-specific demethylase 2B | *kdm2b* | 3.3 | n.d. |
| 1421396_at | proprotein convertase subtilisin/kexin type 1 | *Pcsk1* | 3.3 | n.d. |
| 1449067_at* | solute carrier family 2 (facilitated glucose transporter), member 2 | *Slc2a2* | 3.3 | n.d. |
| 1434326_x_at | coronin, actin binding protein, 2B | *Coro2b* | 3.0 | n.d. |
| 1423690_s_at | G-protein signaling modulator 1 (AGS3-like, C. elegans) | *Gpsm1* | 3.0 | n.d. |
| 1459861_s_at | predicted gene 12397 | *Gm12397* | 3.0 | n.d. |
| 1416868_at | cyclin-dependent kinase inhibitor 2C (p18, inhibits CDK4) | *Cdkn2c* | 2.8 | n.d. |
| 1444139_at | DNA-damage-inducible transcript 4-like | *Ddit4l* | 2.8 | n.d. |
| 1417457_at | similar to cyclin-dependent kinase regulatory subunit 2 (CKS-2); CDC28 protein kinase regulatory subunit 2; predicted gene 15452 | *Loci00044750* | 2.8 | n.d. |
| 1441439_at | urocortin 3 | *Ucn3* | 2.8 | n.d. |
| 1425250_a_at | solute carrier family 14 (urea transporter), member 2 | *Slc14a2* | n.d. | 7.0 |
| 1422826_at | insulin-like growth factor binding protein, acid-labile subunit | *Igfals* | n.d. | 6.1 |
| 1435971_at | regulating synaptic membrane exocytosis 3 | *Rims3* | n.d. | 4.6 |
| 1434709_at | neuron-glia-CAM-related cell adhesion molecule | *Nrcam* | n.d. | 4 |
| 1438211_s_at | D site albumin promoter binding protein | *Dbp* | n.d. | 3.7 |
| 1448929_at | coagulation factor XIII, A1 subunit | *F13a1* | n.d. | 3.7 |
| 1416795_at | crystallin, lambda 1 | *Cryl1* | n.d. | 3.5 |
| 1434548_at | serine incorporator 3 | *Serinc3* | n.d. | 3.2 |
| 1451389_at | DnaJ (Hsp40) homolog, subfamily C, member 24 | *Dnajc24* | n.d. | 3.2 |
| 1416297_s_at | regenerating islet-derived 3 beta | *Reg3b* | n.d. | 3.0 |
| 1440147_at | leucine-rich repeat LGI family, member 2 | *Lgi2* | n.d. | 3.0 |
| 1440859_at | A kinase (PRKA) anchor protein 6 | *Akap6* | n.d. | 2.8 |
| 1439106_at | zinc finger protein 462 | *Zfp462* | n.d. | 2.8 |
| 1434510_at | 3'-phosphoadenosine 5'-phosphosulfate synthase 2 | *Papss2* | n.d. | 2.8 |

**Table S2. Genotyping primers used in this study**

| **Mice** | **Primers** | | **Product size (bp)** |
| --- | --- | --- | --- |
| *MafA* KO (mutant) | Forward | TCTGTTTCAGTCGGATGACCTCCTCCTTGC | 310 |
|  | Reverse | CGATTGTCTGTTGTGCCCAGTC |  |
| Wild-type | Forward | TATGAGGCCTTCCGGGGTCAGAGCTTC | 500 |
|  | Reverse | CGATTGTCTGTTGTGCCCAGTC |  |

**Table S3. Primers used to clone the indicated promoters or to mutagenize the *mPrlr* promoter**

| **Name** | **Primers** | | **Product size (bp)** |
| --- | --- | --- | --- |
| *mPrlr*-1 | Forward | TCTTACGCGTGCTAGCAGGCAAGGAAAGAAAACCGTGAAG | 2359 |
|  | Reverse | CCGGAATGCCAAGCTTTGAGCCCCGTGTAAAATCCAGA |  |
| *mPrlr*-2 | Forward | TCTTACGCGTGCTAGCGTCCACCTCAGCCACCAGAAGC | 1304 |
|  | Reverse | CCGGAATGCCAAGCTTTGAGCCCCGTGTAAAATCCAGA |  |
| *mPrlr*-3 | Forward | TCTTACGCGTGCTAGCTTTCCTCTGACTCCTCCTCCTTCC | 608 |
|  | Reverse | CCGGAATGCCAAGCTTTGAGCCCCGTGTAAAATCCAGA |  |
| *mPrlr*-5 | Forward | TAGTGAACAAGTGCACCGAGTTGAG | 2348 |
|  | Reverse | TGCACTTGTTCACTAGAGGAGGAGTC |  |
| *mPrlr*-6 | Forward | CCGGGAGTACCTTGTCCACCTTTTC | 2348 |
|  | Reverse | ACAAGGTACTCCCGGATCCGTTTCGC |  |
| *mPrlr*-8 | Forward | GAGCAGAATATACAAGTTCTAAGCA | 1742 |
|  | Reverse | TTGTATATTCTGCTCACTGTCTCCAA |  |
| *mPrlr*-9 | Forward | TTGTATATTCTGCTCACTGTCTCCAA | 2094 |
|  | Reverse | TCTATTTTAGTTATCTCTGAACAGGT |  |
| *mPrlr*-11 | Forward | AGCTTCTTGCGGCTGGCGACGGCAGA | 2348 |
|  | Reverse | CAGCCGCAAGAAGCTCAACTCGGTG |  |

**Table S4. TaqMan probes used in this study**

| **Gene symbols** | **Gene** | **ID** | **Species** |
| --- | --- | --- | --- |
| *MafA* | v-maf musculoaponeurotic fibrosarcoma oncogene homolog A (avian) | Mm00845209_s1 | *Mus musculus* |
| *Prlr* | prolactin receptor | Mm00599957_m1 | *Mus musculus* |
| *Ccnd2* | cyclin D2 | Mm00438070_m1 | *Mus musculus* |
| *Slc2a2* | solute carrier family 2 (facilitated glucose transporter), member 2 (Glut2) | Mm00446229_m1 | *Mus musculus* |
| *Vdr* | vitamin D receptor | Mm00437297_m1 | *Mus musculus* |
| *Slc30a8* | solute carrier family 30 (zinc transporter), member 8 (ZnT8) | Mm00555793_m1 | *Mus musculus* |
| *Kdm2b* | lysine (K)-specific demethylase 2B | Mm01194587_m1 | *Mus musculus* |
| *Dapl1* | death associated protein-like 1 | Mm01271524_m1 | *Mus musculus* |
| *Gck* | glucokinase | Mm00439129_m1 | *Mus musculus* |
| *Kcnj11* | potassium inwardly rectifying channel, subfamily J, member 11 | Mm00440050_s1 | *Mus musculus* |
| *Abcc8* | ATP-binding cassette, sub-family C (CFTR/MRP), member 8 | Mm00803450_m1 | *Mus musculus* |
| *MafB* | v-maf musculoaponeurotic fibrosarcoma oncogene family, protein B (avian) | Mm00627481_s1 | *Mus musculus* |
| *Jak2* | Janus kinase 2 | Mm01208489_m1 | *Mus musculus* |
| *Stat5A* | signal transducer and activator of transcription 5A | Mm03053818_s1 | *Mus musculus* |
| *Stat5B* | signal transducer and activator of transcription 5B | Mm00839889_m1 | *Mus musculus* |
| *Ccnd1* | cyclin D1 | Mm00432359_m1 | *Mus musculus* |
| *Ccnd3* | cyclin D3 | Mm01612362_m1 | *Mus musculus* |
| *Cdkn1a* | cyclin-dependent kinase inhibitor 1A (p21) | Mm00432448_m1 | *Mus musculus* |
| *Cdkn1b* | cyclin-dependent kinase inhibitor 1B (p27) | Mm00438168_m1 | *Mus musculus* |
| *Cdkn1c* | cyclin-dependent kinase inhibitor 1C (p57) | Mm00438170_m1 | *Mus musculus* |
| *MafA* | v-maf musculoaponeurotic fibrosarcoma oncogene homolog A (avian) | Rn00845206_s1 | *Rattus norvegicus* |
| *Prlr* | prolactin receptor | Rn00561795_m1 | *Rattus norvegicus* |
| *Ins1* | insulin 1 | Rn02121433_g1 | *Rattus norvegicus* |
| *Ins2* | insulin 2 | Rn01774648_g1 | *Rattus norvegicus* |

**Table S5. Antibodies used in this study**

| **Antigen** | **Species** | **Manufacturer** | **Catalog number** | **Use** |
| --- | --- | --- | --- | --- |
| Insulin | Guinea pig | Millipore | AB3440 | Staining |
| BrdU | Mouse | Dako | M 0744 | Staining |
| Prlr | Rabbit | Santa Cruz | sc-20992 | Immunoblot |
| Stat5 | Rabbit | Santa Cruz | sc-836 | Immunoblot |
| Stat5B | Rabbit | Invitrogen | 71-2500 | Immunoblot/staining |
| Phosphorylated Stat5 | Rabbit | Invitrogen | 71-6900 | Immunoblot |
| Phosphotyrosine | Mouse | BD Transduction | 610000 | Immunoblot |
| Ccnd2 | Rabbit | Santa Cruz | sc-593 | Immunoblot |
| Actin | Mouse | Millipore | MAB1501R | Immunoblot |
| β-Catenin | Mouse | BD Transduction | 610153 | Staining |
